# Explicit Correlation Amplifiers for Finding Outlier Correlations in Deterministic Subquadratic Time

**DOI:** 10.1007/s00453-020-00727-1

**Published:** 2020-06-20

**Authors:** Matti Karppa, Petteri Kaski, Jukka Kohonen, Padraig Ó Catháin

**Affiliations:** 1 Helsinki Institute for Information Technology (HIIT), Espoo, Finland; 2 Department of Computer Science, Aalto University, Espoo, Finland

**Keywords:** Correlation, Derandomization, Outlier, Similarity search, Expander graph

## Abstract

We derandomize Valiant’s (J ACM 62, Article 13, 2015) subquadratic-time algorithm for finding outlier correlations in binary data. This demonstrates that it is possible to perform a deterministic subquadratic-time similarity join of high dimensionality. Our derandomized algorithm gives deterministic subquadratic scaling essentially for the same parameter range as Valiant’s randomized algorithm, but the precise constants we save over quadratic scaling are more modest. Our main technical tool for derandomization is an explicit family of *correlation amplifiers* built via a family of zigzag-product expanders by Reingold et al. (Ann Math 155(1):157–187, 2002). We say that a function $f:\{-1,1\}^d\rightarrow \{-1,1\}^D$ is a *correlation amplifier* with threshold $0\le \tau \le 1$, error $\gamma \ge 1$, and strength *p* an even positive integer if for all pairs of vectors $x,y\in \{-1,1\}^d$ it holds that (i) $|\langle x,y\rangle |<\tau d$ implies $|\langle f(x),f(y)\rangle |\le (\tau \gamma )^pD$; and (ii) $|\langle x,y\rangle |\ge \tau d$ implies $\left (\frac{\langle x,y\rangle }{\gamma d}\right )^pD \le \langle f(x),f(y)\rangle \le \left (\frac{\gamma \langle x,y\rangle }{d}\right )^pD$.

## Introduction

We consider the task of identifying outlier-correlated pairs from large collections of weakly correlated binary vectors in $\{-1,1\}^d$. In more precise terms, we are interested in the following computational problem.

### **Problem 1**

(*Outlier correlations*) We are given as input two sets $X,Y\subseteq \{-1,1\}^d$ with $|X|=|Y|=n$, and two thresholds, the *outlier* threshold $\rho >0$ and the *background* threshold $\tau <\rho$. Our task is to output all *outlier pairs* $(x,y) \in X \times Y$ with $|\langle x,y\rangle |\ge \rho d$, subject to the assumption that at most *q* of the pairs $(x,y)\in X\times Y$ satisfy $|\langle x,y\rangle |>\tau d$.

### *Remark*

This setting of binary vectors and (Pearson) correlation is directly motivated, among others, by the connection to Hamming distance. Indeed, for two vectors $x,y\in \{-1,1\}^d$ we have $\langle x,y\rangle = d-2D_H(x,y)$, where $D_H(x,y)=|\{u=1,2,\ldots ,d:x(u)\ne y(u)\}|$ is the Hamming distance between *x* and *y*.

A naïve way to solve Problem 1 is to compute the $n^2$ inner products $\langle x,y\rangle$ for $(x,y)\in X\times Y$ and filter out everything but the outliers. Our interest is in algorithms that scale *subquadratically* in *n*, when both *d* and *q* are bounded from above by slowly growing functions of *n*. That is, we seek running times of the form $O(n^{2-\epsilon })$ for a constant $\epsilon >0$. Furthermore, we seek to do this without a priori knowledge of *q*.

Running times of the form $O(n^{2-c\rho })$ for a constant $c>0$ are immediately obtainable using techniques such as the seminal *locality-sensitive hashing* of Indyk and Motwani [20] and its variants (see Sect. 1.5). However, such algorithms converge to quadratic running time in *n* unless $\rho$ is bounded from below by a positive constant. Our interest is in algorithms that avoid such a “curse of weak outliers” and run in subquadratic time essentially *independently of the magnitude of* $\rho$, *provided that* $\rho$ *is sufficiently separated from* $\tau$. Such ability to identify weak outliers from large amounts of data is useful, among others, in machine learning from noisy data. Our task can also be seen as a high-dimensional *inner-product similarity join* on a large number of weakly similar attributes.

One strategy to circumvent the curse of weak outliers is to pursue the following intuition: (1) partition the input vectors into *buckets* of at most *s* vectors each, (2) aggregate each bucket into a single vector by taking the vector sum, and (3) compute the inner products between the $\lceil n/s\rceil \times \lceil n/s\rceil$ pairs of aggregate vectors. With sufficient separation between $\tau$ and $\rho$, at most *q* of these inner products between aggregates will be large, and every outlier pair is discoverable among the at most $s\times s$ input pairs that correspond to each large inner product of aggregates. Furthermore, a strategy of this form is oblivious to *q* until we actually start searching inside the buckets, which enables adjusting $\rho$ and $\tau$ based on the number of large aggregate inner products.

### Randomized Amplification

Such bucketing strategies have been studied before with the help of randomization. In 2012, Valiant [36] presented a breakthrough algorithm that, before bucketing, replaces each input vector with a randomly subsampled^1^ version of its $p\mathrm {th}$ Kronecker power. Because of the tensor-power identity

1 $$\begin{aligned} \langle x^{\otimes p},y^{\otimes p}\rangle =\langle x,y\rangle ^p, \end{aligned}$$

the ratio between outlier and background correlations gets *amplified* to essentially its $p\mathrm {th}$ power, assuming that the sample is large enough so that sufficient concentration bounds hold with high probability. This amplification makes the outliers stand out from the background even after bucketing, which enables detection in subquadratic time using fast matrix multiplication.

A subset of the present authors [23] further improved on Valiant’s algorithm by a modified sampling scheme that *simultaneously* amplifies and aggregates the input by further use of fast matrix multiplication. With this improvement, Problem 1 can be solved in subquadratic time if the logarithmic ratio $\log _\tau \rho = (\log \rho )/(\log \tau )$ is bounded from above by a constant less than 1. Also this improved algorithm relies on randomization.

### Explicit Amplification

In this paper we seek *deterministic* subquadratic algorithms. As with the earlier randomized algorithms, we seek to map the *d*-dimensional input vectors to a higher dimension *D* so that inner products are sufficiently amplified in the process. Towards this end, we are interested in explicit functions $f : \{-1,1\}^d \rightarrow \{-1,1\}^D$ that approximate the tensor-power identity (1).

#### **Definition 1**

(*Correlation amplifier*) Let *d*, *D* and *p* be positive integers, with *p* even, and let $0\le \tau \le 1$ and $\gamma \ge 1$. A function $f : \{-1,1\}^d \rightarrow \{-1,1\}^D$ is a *correlation amplifier* with parameters $(d,D,p,\tau ,\gamma )$ if for all pairs of vectors $x,y\in \{-1,1\}^d$ we have

2 $$\begin{aligned}&\text { if } \left |\langle x,y\rangle \right | < \tau d \text {, then } \left |\langle f(x),f(y)\rangle \right | \le (\tau \gamma )^pD \,;\,\, \text {and} \end{aligned}$$

3 $$\begin{aligned}&\text { if } \left |\langle x,y\rangle \right |\ge \tau d \text {, then } \left( \frac{\langle x,y\rangle }{\gamma d} \right) ^{p}D \le \langle f(x),f(y)\rangle \le \left( \frac{\gamma \langle x,y\rangle }{d} \right) ^{p}D. \end{aligned}$$

#### *Remark*

A correlation amplifier *f* guarantees by (2) that correlations below $\tau$ in absolute value stay bounded; and by (3) that correlations at least $\tau$ in absolute value *become positive* and are governed by the two-sided approximation with multiplicative error $\gamma \ge 1$. In particular, (3) implies that correlations at least $\tau$ cannot mask outliers under bucketing because all such correlations get positive sign under amplification.

It is immediate that correlation amplifiers exist. For example, take $f(x)=x^{\otimes p}$, with *p* even, to obtain a correlation amplifier with $D=d^p$, $\tau =0$, and $\gamma =1$ by (1). For our present purposes, however, we seek correlation amplifiers with *D* substantially smaller than $d^p$. Furthermore, we seek constructions that are *explicit* in the strong^2^ form that there exists a deterministic algorithm that computes any individual coordinate of *f*(*x*) in time $\mathrm {poly}(\log D,p)$ by accessing $\mathrm {poly}(p)$ coordinates of a given $x\in \{-1,1\}^d$. In what follows explicitness always refers to this strong form.

### Our Results

The main result of this paper is that sufficiently powerful explicit amplifiers exist to find outlier correlations in deterministic subquadratic time.

#### **Theorem 1**

(Explicit amplifier family) *There exists an explicit correlation amplifier* $f:\{-1,1\}^{d}\rightarrow \{-1,1\}^{2^K}$ *with parameters* $(d,2^K,2^\ell ,\tau ,\gamma )$ *whenever* $0<\tau <1$, $\gamma >1$, *and* $d,K,\ell$ *are positive integers with*

4 $$\begin{aligned} 2^K\ge d \left (2^{10} \right (1-\gamma ^{-1/2}\left )^{-1}\right )^{20\ell +1} \left (\frac{\gamma }{\tau }\right )^{60\,\cdot \,2^{\ell }} . \end{aligned}$$

As a corollary we obtain a deterministic algorithm for finding outlier correlations in subquadratic time using bucketing and fast matrix multiplication. Let us write $\alpha$ for the limiting exponent of rectangular integer matrix multiplication. That is, for all constants $\eta >0$ there exists an algorithm that multiplies an $m\times \lfloor m^\alpha \rfloor$ integer matrix with an $\lfloor m^{\alpha }\rfloor \times m$ integer matrix in $O(m^{2+\eta })$ arithmetic operations. In particular, it is known that $0.3<\alpha \le 1$ [25].

#### **Theorem 2**

(Deterministic subquadratic algorithm for outlier correlations) *For any constants* $0<\epsilon <1$,  $0<\tau _\mathrm {max}<1$,  $0<\delta <\alpha$, *and* $C>60$, *there exists a deterministic algorithm that solves a given instance of Problem* 1*in time*

5 $$\begin{aligned} O\left (n^{2-\frac{0.99\epsilon (\alpha -\delta )}{4C+1}}+ qn^{\delta +\frac{1.99\epsilon (\alpha -\delta )}{4C+1}}\right ) \end{aligned}$$

*assuming that the parameters* $n,d,\rho ,\tau$ *satisfy the following three constraints*

1. $$d\le n^{\delta }$$,
2. $$c_1 n^{-c_2} \le \tau \le \tau _\mathrm {max}$$, *where* $$c_1 = \tau _\mathrm {max}^{-\epsilon /100{,}000}$$, $$c_2 = \left( 1-\frac{0.99\epsilon }{4C+1}\right) \frac{\alpha -\delta }{C}$$, *and*
3. $$\log _\tau \rho \le 1-\epsilon$$.

#### *Remark*

In contrast to LSH-based techniques discussed previously, the running time (5) remains subquadratic regardless of the magnitude of $\rho$ provided that the conditions of Theorem 2 are satisfied, particularly the separation of $\rho$ and $\tau$ in condition 3.^3^ The constants in (4) and (5) have not been optimized beyond our desired goal of obtaining deterministic subquadratic running time when *d* and *q* are bounded by slowly growing functions of *n*. In particular, (5) gives substantially worse subquadratic running times compared with the existing randomized strategies [23, 36]. The algorithm in Theorem 2 needs no *a priori* knowledge of *q* and is oblivious to *q* until it starts searching inside the buckets.

### Overview and Discussion of Techniques

A straightforward application of the probabilistic method (Lemma 12) establishes that low-dimensional correlation amplifiers can be obtained by subsampling uniformly at random the dimensions of the tensor power $x^{\otimes p}$ as long as the sample size *D* is large enough. Thus, in essence our Theorem 1 amounts to *derandomizing* such a subsampling strategy by presenting an explicit sample that is, up to the error bounds (2) and (3), indistinguishable from the “perfect” amplifier $x\mapsto x^{\otimes p}$ under taking of inner products.

The construction underlying Theorem 1 amounts to an $\ell$-fold composition of explicit *squaring* amplifiers ($p=2$) with increasingly strong control on the error ($\gamma$) and the interval of amplification ($[\tau ,1]$) at each successive composition. Towards this end, we require a flexible explicit construction of squaring amplifiers with strong control on the error and the interval. We obtain such a construction from an explicit family of expander graphs (Lemma 2) obtainable from the explicit zigzag-product constructions of Reingold et al. [34]. In particular, the key to controlling the error and the interval is that the expander family gives *Ramanujan-like*^4^ concentration $\lambda /{\varDelta }\le 16{\varDelta }^{-1/4}$ of the normalized second eigenvalue $\lambda /{\varDelta }$ by increasing the degree ${\varDelta }$. In essence, since we are working with $\{-1,1\}$-valued vectors, by increasing the degree we can use the Expander Mixing Lemma (Lemma 1) and the Ramanujan-like concentration to control (Lemma 4) how well the restriction $x^G$ to the edges of an expander graph *G* approximates the full tensor square $x^{\otimes 2}$ under taking of inner products.

Our construction has been motivated by the paradigm of *gradually increasing independence* [9, 14, 15, 21] in the design of pseudorandom generators. Indeed, we obtain the final amplifier gradually by successive squarings, taking care that the degree ${\varDelta }_i$ of the expander that we apply in each squaring $i=0,1,\ldots ,\ell -1$ increases with a similar squaring schedule given by (11) and (15) to simultaneously control the error and the interval, and to bound the output dimension roughly by the square of the degree of the last expander in the sequence. Here the term “gradual” is not particularly descriptive since growth under successive squaring amounts to *doubly* exponential growth in the number of squarings. Yet such growth *can* be seen as gradual and controlled in the following sense: we obtain strong amplification compared with the final output dimension precisely because the first $\ell -1$ squarings “come for free” as ${\varDelta }_0{\varDelta }_1\cdots {\varDelta }_{\ell -2}$ is (up to low-order multiplicative terms) no more than ${\varDelta }_{\ell -1}^2$, essentially because we are taking the sum of powers of 2 in the exponent.

The analogy with pseudorandom generators can in fact be pushed somewhat further. Namely, a correlation amplifier can be roughly seen as a pseudorandom generator that by (3) seeks to fool a “truncated family of uniform combinatorial rectangles” with further control requested by (2) below the truncation threshold $\tau$. To see the rough analogy, let $z\in \{-1,1\}^d$ be the Hadamard product of the vectors $x,y\in \{-1,1\}^d$ and observe that (3) seeks to approximate (with multiplicative error) the expectation of a uniform random entry in the $d^p$-length Kronecker power $z^{\otimes p}$ by instead taking the expectation over an explicit *D*-dimensional sample given by *f*. The Kronecker power $z^{\otimes p}$ is a uniform special case (with $z=z_1=z_2=\cdots =z_p$) of a “combinatorial rectangle” formed by a Kronecker product $z_1\otimes z_2\otimes \cdots \otimes z_p$, and truncation means that we only seek approximation in cases where $|\sum _{u=1}^d z(u)|\ge \tau d$, and accordingly want constructions that take this truncation into account—that is, we do not seek to fool all combinatorial rectangles and accordingly want stronger control on the dimension *D* (that is, the “seed length” $\log D$).

For a review of the state of the art in pseudorandom generators we refer to Gopalan et al. [14] and Kothari and Meka [24]. Our goal to obtain a small output dimension *D* roughly corresponds to optimizing the seed length of a pseudorandom generator.

While our explicit construction (4) does not reach the exact output dimension obtainable by Lemma 12, it should be observed that in our parameter range of interest (with $\gamma >1$ a constant and $0<\tau \le \tau _{\mathrm {max}}$ for a constant $0<\tau _{\mathrm {max}}<1$), both (4) and (32) are of the form $D\ge d\tau ^{-{\varTheta }(p)}$; only the constants hidden by the asymptotic notation differ between the explicit and nonconstructive bounds. Moreover, using results of Alon [4] we show a *lower bound* (Lemma 17) on the output dimension *D* of any correlation amplifier: namely, that $D \ge \frac{1}{5} (\frac{1}{\gamma \tau })^p$, when *p* is in the range governed by $(\gamma \tau )^p \le 1/100$ and $p \le \frac{(\log e)\tau ^2d}{8\log (\frac{1}{\gamma \tau })}$. Thus, viewed as a pseudorandom generator with “seed length” $\log D$, Theorem 1 essentially does not admit improvement except possibly at the multiplicative constants.

### Related Work and Applications

Problem 1 is a basic problem in data analysis and machine learning admitting many extensions, restrictions, and variants. A large body of work exists studying *approximate near neighbour search* via techniques such as locality-sensitive hashing (e.g. [5–7, 13, 20, 29, 30]), with recent work aimed at derandomization (see Pagh [31] and Pham and Pagh [33]) and resource tradeoffs (see Kapralov [22]) in particular. However, these techniques enable subquadratic scaling in *n* only when $\rho$ is bounded from below by a positive constant, whereas the algorithm in Theorem 2 remains subquadratic even in the case of weak outliers when $\rho$ tends to zero with increasing *n*, as long as $\rho$ and $\tau$ are separated. Ahle et al. [1] show that subquadratic scaling in *n* is not possible for $\log _\tau \rho =1-o(1/\sqrt{\log n})$ unless both the Orthogonal Vectors Conjecture and the Strong Exponential Time Hypothesis [19] fail.

In the context of databases and information retrieval, inner product is one of the widely used metrics for *similarity join* [1]. Apart from low-dimensional approaches such as trees that partition the space in one dimension at a time, existing scalable methods for high-dimensional data employ randomization, leading to the possibility of false negatives (missed pairs) and false positives (spurious pairs). Recently Pagh [31] and Pham and Pagh [33] eliminated false negatives completely; however, their method still has a random running time. We present here a completely deterministic solution.

In small dimensions, Alman and Williams [3] present a randomized algorithm that finds *exact* Hamming-near neighbours in a batch-query setting analogous to Problem 1 in subquadratic time in *n* when the dimension is constrained to $d=O(\log n)$. Recently, Chan and Williams [10] show how to derandomize related algorithm designs; also, Alman et al. [2] derandomize the probabilistic polynomials for symmetric Boolean functions used in [3], achieving deterministic subquadratic batch queries in small dimensions.

One special case of Problem 1 is the problem of learning a weight 2 parity function in the presence of noise, or *the light bulb problem*.

#### **Problem 2**

(*Light bulb problem, Valiant* [37]) Suppose we are given as input a parameter $0<\rho <1$ and a set of *n* vectors in $\{-1,1\}^d$ such that one *planted* pair of vectors has inner product at least $\rho d$ in absolute value, and all other $n-2$ vectors are chosen independently and uniformly at random. Our task is to find the planted pair among the *n* vectors.

#### *Remark*

From e.g. the Hoeffding bound (7) it follows that there exists a constant *c* such that when $d\ge c\rho ^{-2}\log n$ the planted pair is with high probability (as *n* increases) the unique pair in the input with the maximum absolute correlation.

For a problem whose instances are drawn from a random ensemble, we say that an algorithm solves *almost all* instances of the problem if the probability of drawing an instance where the algorithm fails tends to zero as *n* increases.

Paturi et al. [32], Dubiner [11], and May and Ozerov [27] present randomized algorithms that can be used to solve almost all instances of the light bulb problem in subquadratic time if we assume that $\rho$ is bounded from below by a positive constant; if $\rho$ tends to zero these algorithms converge to quadratic running time in *n*.

Valiant [36] showed that a randomized algorithm can identify the planted correlation in subquadratic time on almost all inputs even when $\rho$ tends to zero as *n* increases. As a corollary of Theorem 2, we can derandomize Valiant’s design and still retain subquadratic running time (but with a worse constant) for almost all inputs, except for extremely weak planted correlations with $\rho \le n^{-{\varOmega }(1)}$ that our amplifier is not in general able to amplify with sufficiently low output dimension to enable an overall subquadratic running time.

#### **Corollary 1**

(Deterministic subquadratic algorithm for the light bulb problem) *For any constants* $0<\delta <\alpha$, $C>60$, $0<\rho _{\mathrm {max}}<1$, *and* $\kappa >1$, *there exists a deterministic algorithm that solves almost all instances of Problem* 2*in time*

$$\begin{aligned} O\left (n^{2-\frac{0.99(1-1/\kappa )(\alpha -\delta )}{4C+1}}\right ) \end{aligned}$$

*assuming the parameters* $n,d,\rho$ *satisfy the two constraints*

1. $$5\rho ^{-2\kappa }\log n\le d \le n^\delta$$ *and*
2. $$c_1n^{-c_2/\kappa }\le \rho \le \rho _{\max }$$,

*where* $$c_1 = \rho _\mathrm {max}^{-\kappa \epsilon /100{,}000}$$ *and* $$c_2 = \left( 1-\frac{0.99(1-1/\kappa )}{4C+1}\right) \frac{\alpha -\delta }{C}$$.

Corollary 1 extends to parity functions of larger (constant) weight in the presence of noise (cf. [16, 23, 36]). This generalized version of the problem is as follows.

#### **Problem 3**

(*Learning parity with noise*) Let $S\subseteq [v]$ with $|S| = k$ be the *support* of a parity function and $0< \eta < 1$ the noise level. Our task is to determine the set *S* by drawing independent random examples (*x*, *y*) such that $x\in \{-1,1\}^v$ is chosen uniformly at random, and the *label* $y\in \{-1,1\}$ is $y = z\prod _{\ell \in S} x(\ell )$ where $z \in \{-1,1\}$ is an independent random variable with $\Pr (z = -1) = \eta$.

With no information on *k*, the trivial solution is to enumerate all $2^v$ subsets of [*v*] to locate the support *S*. Blum et al. [8] provide a non-trivial solution which runs in time and sample complexity $\text {poly}\left (|1-2\eta |^{2^a},2^b\right )$ for any positive integers *a*, *b* with $ab\ge v$; this is $2^{O(v/\log v)}$ when $\eta \ne 1/2$ is a constant independent of *v*. If we assert that *k* is a constant independent of *v*, the trivial complexity drops from exponential to $v^k$, and non-trivial speed-ups seek to lower the coefficient 1 of *k* in the exponent. Randomized solutions for constant *k* include Valiant’s breakthrough algorithm [36] and our subsequent randomized improvement [23] which runs in time ${\tilde{O}}(v^{\frac{\omega +\epsilon }{3}k}|1-2\eta |^{-\frac{8\omega }{9\epsilon }-\frac{4}{3}})$ for any constant $0<\epsilon <\omega /3$.

Our present contribution is a deterministic algorithm for learning constant-weight parity functions with noise. Our interest is in the case where the noise level $\eta$ approaches 1/2, and accordingly we assume that $|1-2\eta |$ is bounded from above by a constant less than 1. We say that a deterministic algorithm *solves almost all instances* of Problem 3 if the probability of drawing an instance on which the algorithm fails tends to zero as *v* increases.^5^

#### **Corollary 2**

(Deterministic algorithm for learning parity with noise) *For all constants* $0< \delta < \alpha$, $C>60$, $\xi > 1$, $0<\theta < 1$, *there exists a constant* $k_0$ *and a deterministic algorithm that for all constants* $k\ge k_0$ *draws* *d* *examples and finds the support of almost all instances of Problem* 3*in time*

6 $$\begin{aligned} O\left ( v^{k\left (1-0.245025(\alpha -\delta )^2(1-1/\xi )^2(1+4C)^{-2}\right )} \right ) , \end{aligned}$$

*assuming the parameters* $v,d,\eta$ *satisfy the constraints*

1. $$d \ge \frac{6k}{|1-2\eta |^{2(\xi ^2+1)}(1-\theta ^{\xi -1})^2}\log v$$, *and*
2. $$c_1v^{-c_2\xi ^{-2}k/2} \le |1-2\eta | \le \theta$$,

*where* $$c_1 = \theta ^{-(1-1/\xi )/100{,}000}$$ *and* $$c_2 = \left( 1-\frac{0.99(1-1/\xi )}{4C+1}\right) \frac{\alpha -\delta }{C}$$.

Algorithms for learning parity functions enable extensions to further classes of Boolean functions such as sparse juntas and DNFs (cf. [12, 28, 36]).

## Preliminaries

All vectors in this paper are integer-valued. For a vector $x\in {\mathbb {Z}}^d$ we denote the entry $u=1,2,\ldots ,d$ of *x* by *x*(*u*). For two vectors $x,y\in {\mathbb {Z}}^d$ we write $\langle x,y\rangle =\sum _{u=1}^d x(u)y(u)$ for the inner product of *x* and *y*. We write $\log$ for the logarithm with base 2 and $\ln$ for the logarithm with base $\exp (1)$.

In our proofs, we need the following bound due to Hoeffding [17, Theorem 2] which provides an exponentially small upper bound on the deviation of a sum of bounded independent random variables from its expectation.

### **Theorem 3**

(Hoeffding [17, Theorem 2]) *Let* $Z_1,Z_2,\ldots ,Z_D$ *be independent random variables satisfying* $\ell _i \le Z_i \le u_i$ *for all* $1 \le i \le D$, *and let* $Z = \sum _{i=1}^D Z_i$. *Then, for all* $c > 0$, *the following holds:*

7 $$\begin{aligned} {\text {Pr}} \left( Z - {\mathrm {E}}[Z] \ge c \right) \le \exp \left( -\frac{2c^2}{\sum _{i=1}^D(u_i-\ell _i)^2} \right) . \end{aligned}$$

## Explicit Amplifiers by Approximate Squaring

This section proves Theorem 1. We start with preliminaries on expanders, show an approximate squaring identity using expander mixing, and then rely on repeated approximate squaring for our main construction. The proof is completed by some routine preprocessing.

### Preliminaries on Expansion and Mixing

We work with undirected graphs, possibly with self-loops and multiple edges. A graph *G* is ${\varDelta }$-*regular* if every vertex is incident to exactly ${\varDelta }$ edges, with each self-loop (if present) counting as one edge. Suppose that *G* is ${\varDelta }$-regular with vertex set *V*, and let *L* be a set of ${\varDelta }$ labels such that the ${\varDelta }$ edge-ends incident to each vertex have been labeled with unique labels from *L*. The *rotation map* $\mathrm {Rot}_G:V\times L\rightarrow V\times L$ is the bijection such that for all $u\in V$ and $i\in L$ we have $\mathrm {Rot}_G(u,i)=(v,j)$ if the edge incident to vertex *u* and labeled with *i* at *u* leads to the vertex *v* and has the label *j* at *v*.

For $S,T\subseteq V(G)$, let us write *E*(*S*, *T*) for the set of edges of *G* with one end in *S* and the other end in *T*. Suppose that *G* has *D* vertices and let $\lambda _1,\lambda _2,\ldots ,\lambda _D$ be the eigenvalues of the adjacency matrix of *G* with $|\lambda _1|\ge |\lambda _2|\ge \cdots \ge |\lambda _D|$. Let us say that a graph *G* is a $(D,{\varDelta },\lambda )$-*graph* if *G* has *D* vertices, *G* is ${\varDelta }$-regular, and $|\lambda _2|\le \lambda$. For an excellent survey on expansion and expander graphs, we refer to Hoory et al. [18].

#### **Lemma 1**

(Expander mixing lemma, [18, Lemma 2.5]) *For all* $S,T\subseteq V(G)$ *we have*

$$\begin{aligned} \left ||E(S,T)|-\frac{{\varDelta }|S||T|}{D}\right | \le \lambda \sqrt{|S||T|}. \end{aligned}$$

We work with the following family of graphs obtained from the zig-zag product of Reingold et al. [34]. In particular Lemma 2 gives us $\lambda /{\varDelta }\le 16{\varDelta }^{-1/4}$, which will enable us to control relative inner products by increasing ${\varDelta }$.

#### **Lemma 2**

*For all integers* $t\ge 1$ *and* $b\ge 10$ *there exists a* $(2^{16bt},2^{4b},16\cdot 2^{3b})$-*graph whose rotation map can be evaluated in time* $\mathrm {poly}(b,t)$.^6^

#### *Proof*

See “Appendix”. $$\square$$

### Main Construction

The main objective of this section is to prove the following lemma, which we will then augment to Theorem 1 by routine preprocessing of the input dimension.

#### **Lemma 3**

(Repeated approximate squaring) *There exists an explicit correlation amplifier* ${\hat{f}}:\{-1,1\}^{2^k}\rightarrow \{-1,1\}^{2^K}$ *with parameters* $(2^k,2^K,2^\ell ,\tau _0,\gamma _0)$ *whenever* $0<\tau _0<1$, $\gamma _0>1$, *and* $k,K,\ell$ *are positive integers with*

8 $$\begin{aligned} 2^K\ge 2^k \left (2^{10}\right (1-\gamma _0^{-1}\left )^{-1}\right )^{20\ell } \left (\frac{\gamma _0}{\tau _0}\right )^{40\,\cdot \,2^{\ell }-20} . \end{aligned}$$

*Approximate squaring via expanders* For a vector $x\in \{-1,1\}^D$, let us write $x^{\otimes 2}\in \{-1,1\}^{D^2}$ for the Kronecker product of *x* with itself. Our construction for correlation amplifiers will rely on approximating the *squaring identity*

$$\begin{aligned} \langle x^{\otimes 2},y^{\otimes 2}\rangle = \langle x,y\rangle ^2, \end{aligned}$$

for vectors in $\{-1, 1\}^{D}$. In more precise terms, let *G* be a $(D,{\varDelta },\lambda )$-graph and let $x^G\in \{-1,1\}^{{\varDelta }D}$ be a vector that contains each coordinate *x*(*u*)*x*(*v*) of $x^{\otimes 2}$ with $(u,v)\in V(G)\times V(G)$ exactly once for each edge of *G* that joins the vertex *u* to the vertex *v*. Equivalently, let $\mathrm {Rot}_G:V\times L\rightarrow V\times L$ be a rotation map for *G*, and define $x^G$ for all $u\in V$ and all $i\in L$ by $x^G(u,i)=x(u)x(v)$ where $v\in V$ is given by $\mathrm {Rot}_G(u,i)=(v,j)$. In particular, $x^G$ has exactly ${\varDelta }D$ coordinates.

#### **Lemma 4**

(Approximate squaring) *For all* $x,y\in \{-1, 1\}^D$ *we have*

$$\begin{aligned} \left| \langle x^G,y^G \rangle -\frac{{\varDelta }}{D}\langle x^{\otimes 2},y^{\otimes 2}\rangle \right| \le 2\lambda D. \end{aligned}$$

#### *Proof*

Let $S=\{u\in V(G):x(u)=y(u)\}$ and let us write ${\bar{S}}=V(G){\setminus } S$. Since *x*, *y* are $\{-1,1\}$-valued, we have

$$\begin{aligned} \langle x^G, y^G \rangle = |E(S,S)|+|E({\bar{S}},{\bar{S}})|-|E(S,{\bar{S}})|-|E({\bar{S}},S)|. \end{aligned}$$

Observing that

$$\begin{aligned} |S|^2+|{\bar{S}}|^2-|S||{\bar{S}}|-|{\bar{S}}||S| =\left (2|S|-D\right )^2 =\langle x,y\rangle ^2 =\langle x^{\otimes 2},y^{\otimes 2}\rangle \end{aligned}$$

and applying Lemma 1 four times, we have

$$\begin{aligned} \left| \langle x^G,y^G \rangle -\frac{{\varDelta }}{D}\langle x^{\otimes 2},y^{\otimes 2}\rangle \right| \le \lambda \left (D+2\sqrt{|S|(D-|S|)}\right ) \le 2\lambda D. \end{aligned}$$

$$\square$$

*The amplifier function* We now construct an amplifier function ${\hat{f}}$ that uses $\ell$ approximate squarings, $\ell \ge 1$, with the graphs drawn from the graph family in Lemma 2. Accordingly, we assume that all vectors have lengths that are positive integer powers of 2.

The input $x={\tilde{x}}_0\in \{-1,1\}^{d_0}$ to the amplifier has dimension $d_0=2^k$ for a positive integer *k*. For $i=0,1,\ldots ,\ell -1$, suppose we have the vector ${\tilde{x}}_i\in \{-1,1\}^{d_i}$. Let $b_i$ be a positive integer whose value will be fixed later. Let $t_i$ be the unique positive integer with

$$\begin{aligned} d_i\le D_i=2^{16b_it_i}<2^{16b_i}d_i. \end{aligned}$$

Note in particular that $d_i$ divides $D_i$ since $d_i$ is a power of 2. Let $G_i$ be a $(2^{16b_it_i}, 2^{4b_i}, 16\cdot 2^{3b_i})$-graph from Lemma 2. Take $D_i/d_i$ copies of ${\tilde{x}}_i$ to obtain the vector $x_i\in \{-1,1\}^{D_i}$. Let ${\tilde{x}}_{i+1}=x_i^{G_i}\in \{-1,1\}^{d_{i+1}}$ with $d_{i+1}={\varDelta }_iD_i$ and ${\varDelta }_i=2^{4b_i}$. The amplifier outputs ${\hat{f}}(x)={\tilde{x}}_\ell$ with ${\tilde{x}}_\ell \in \{-1,1\}^{d_\ell }$.

Since the graph family in Lemma 2 admits rotation maps that can be computed in time $\mathrm {poly}(b,t)$, we observe that ${\hat{f}}$ is explicit. Indeed, from the construction it is immediate that to compute any single coordinate of ${\hat{f}}(x)$ it suffices to (i) perform in total $2^{\ell -1-i}$ evaluations of the rotation map of the graph $G_i$ for each $i=0,1,\ldots ,\ell -1$, and (ii) access at most $2^\ell$ coordinates of *x*. Since $b_it_i=O(\log d_\ell )$ for all $i=0,1,\ldots ,\ell -1$, we have that we can compute any coordinate of ${\hat{f}}(x)$ in time $\mathrm {poly}(\log d_\ell ,2^\ell )$ and accessing at most $2^\ell$ coordinates of *x*.

*Parameterization and analysis* Fix $\tau _0>0$ and $\gamma _0>1$. To parameterize the amplifier (that is, it remains to fix the values $b_i$), let us track a pair of vectors as it proceeds through the $\ell$ approximate squarings for $i=0,1,\ldots ,\ell -1$.

We start by observing that copying preserves *relative* inner products. That is, for any pair of vectors ${\tilde{x}}_i,{\tilde{y}}_i\in \{-1,1\}^{d_i}$ we have $\langle {\tilde{x}}_i,{\tilde{y}}_i\rangle =\nu _i d_i$ if and only if $\langle x_i, y_i \rangle =\nu _i D_i$ for $0\le \nu _i\le 1$.

An easy manipulation of Lemma 4 using the parameters in Lemma 2 gives us additive control over an approximate squaring via

9 $$\begin{aligned} \nu _i^2 - 32{\varDelta }_i^{-1/4} \le \nu _{i+1} \le \nu _i^2 + 32{\varDelta }_i^{-1/4}. \end{aligned}$$

For all inner products that are in absolute value above a threshold, we want to turn this additive control into multiplicative control via

10 $$\begin{aligned} \nu _i^2\gamma _0^{-1} \le \nu _{i+1} \le \nu _i^2\gamma _0. \end{aligned}$$

Let us insist this multiplicative control holds whenever $|\nu _i|\ge \tau _i$ for the threshold parameter $\tau _i$ defined for all $i=0,1,\ldots ,\ell -1$ by

11 $$\begin{aligned} \tau _{i+1}=\gamma _0^{-1}\tau _i^2. \end{aligned}$$

Enforcing (10) via (9) at the threshold, let us assume that

12 $$\begin{aligned} \tau _i^2\gamma _0^{-1}\le \tau _i^2-32{\varDelta }_i^{-1/4}. \end{aligned}$$

The next lemma confirms that assuming (12) gives two-sided control of inner products which is retained to the next approximate squaring. The following lemma shows that small inner products remain small.

#### **Lemma 5**

*If* $\tau _i\le |\nu _i|$, *then* $\nu _i^2\gamma _0^{-1}\le \nu _{i+1}\le \nu _i^2\gamma _0$ *and* $\tau _{i+1}\le \nu _{i+1}$.

#### *Proof*

From (9) and (12), we have

13 $$\begin{aligned} \left| \nu _{i+1}-\nu _i^2\right| \le 32{\varDelta }_i^{-1/4} \le (1-\gamma _0^{-1})\tau _i^2 \le (1-\gamma _0^{-1})\nu _i^2. \end{aligned}$$

Observe that $1-\gamma _0^{-1}\le \gamma _0-1$. Thus, from (13) we conclude that

$$\begin{aligned} \nu _{i+1} \le \nu _i^2+(1-\gamma _0^{-1})\nu _i^2 \le \nu _i^2+(\gamma _0-1)\nu _i^2 = \gamma _0\nu _i^2. \end{aligned}$$

In the converse direction, from (13) and (11) we conclude that

$$\begin{aligned} \nu _{i+1} \ge \nu _i^2-(1-\gamma _0^{-1})\nu _i^2 \ge \gamma _0^{-1}\nu _i^2 \ge \gamma _0^{-1}\tau _i^2 =\tau _{i+1}. \end{aligned}$$

$$\square$$

#### **Lemma 6**

*If* $$|\nu _i|<\tau _i$$, *then* $$|\nu _{i+1}|\le \tau _i^2\gamma _0$$.

#### *Proof*

From (9) and (12), we have

14 $$\begin{aligned} \left| \nu _{i+1}-\nu _i^2\right| \le 32{\varDelta }_i^{-1/4} \le (1-\gamma _0^{-1})\tau _i^2. \end{aligned}$$

Since $1-\gamma _0^{-1}\le \gamma _0-1$, from (14) we conclude that

$$\begin{aligned} |\nu _{i+1}| \le \nu _i^2+(1-\gamma _0^{-1})\tau _i^2 \le \tau _i^2+(\gamma _0-1)\tau _i^2 = \gamma _0\tau _i^2. \end{aligned}$$

$$\square$$

Let us now make sure that (12) holds. Solving for ${\varDelta }_i$ in (12), we have

15 $$\begin{aligned} {\varDelta }_i\ge \left( 32(1-\gamma _0^{-1})^{-1}\tau _{i}^{-2}\right) ^{4}. \end{aligned}$$

In particular, we can make sure that (15) and hence (12) holds by simply choosing a large enough ${\varDelta }_i$ (that is, a large enough $b_i$).

Before proceeding with the precise choice of $b_i$ for $i=0,1,\ldots ,\ell -1$, let us analyze the input–output relationship of the amplifier ${\hat{f}}$ using Lemmas 5 and 6. Let $x,y\in \{-1,1\}^{d_0}$ be two vectors given as input with $\langle x,y\rangle =\nu _0d_0$. The outputs ${\hat{f}}(x),{\hat{f}}(y)\in \{-1,1\}^{d_\ell }$ then satisfy $\langle {\hat{f}}(x),{\hat{f}}(y)\rangle =\nu _\ell d_\ell$, where the following two lemmas control $\nu _\ell$ via $\nu _0$.

#### **Lemma 7**

*If* $$|\nu _0|\ge \tau _0$$, *then* $$\nu _0^{2^\ell } \gamma _0^{-2^\ell +1} \le \nu _\ell \le \nu _0^{2^\ell }\gamma _0^{2^\ell -1}$$.

#### *Proof*

Use induction on *i*, where Lemma 5 gives the inductive step. $\square$

#### **Lemma 8**

*If* $$|\nu _0|<\tau _0$$, *then* $$|\nu _\ell |\le \tau _0^{2^\ell }\gamma _0^{2^\ell -1}$$.

#### *Proof*

From (11) we have $\tau _i=\tau _0^{2^i}\gamma _0^{-2^i+1}$. Let us show by induction on *i* that $|\nu _i|\le \tau _0^{2^i}\gamma _0^{2^i-1}$. The base case $i=0$ is immediate. For $i\ge 1$, there are two cases to consider. First suppose that $|\nu _i|<\tau _i$. Then, by Lemma 6 we have $|\nu _{i+1}|\le \tau _i^2\gamma _0\le \tau _0^{2^{i+1}} \gamma _0^{-2^{i+1}+3}\le \tau _0^{2^{i+1}}\gamma _0^{2^{i+1}-1}$ since $\gamma _0>1$. Next suppose that $|\nu _i|\ge \tau _i$. Then, by Lemma 5 we have $|\nu _{i+1}|\le \nu _i^2\gamma _0\le \tau _0^{2^{i+1}}\gamma _0^{2^{i+1}-1}$. $\square$

Since $\gamma _0>1$, from Lemmas 7 and 8 it now follows that ${\hat{f}}$ meets the required amplification constraints (2) and (3) with $p=2^\ell$, $\tau =\tau _0$, and $\gamma =\gamma _0$.

Let us now complete the parameterization and derive an upper bound for $d_\ell$. For each $i=0,1,\ldots ,\ell -1$, take $b_i$ to be the smallest nonnegative integer so that $b_i\ge 10$ and ${\varDelta }_i=2^{4b_i}$ satisfies (15). Since $D_i\le 2^{16b_i}d_i={\varDelta }_i^4d_i$, we have $d_{i+1} = {\varDelta }_{i}D_{i} \le {\varDelta }_{i}^{5}d_{i}$, and hence

$$\begin{aligned} d_{\ell } \le \left( {\varDelta }_{\ell -1}{\varDelta }_{\ell -2}\cdots {\varDelta }_0\right) ^5d_{0}. \end{aligned}$$

Recall that $d_0=2^k$. From (15) we have that

$$\begin{aligned} {\varDelta }_i =2^{4b_i} \le \max \left (2^{40},2^4\left (32(1-\gamma _0^{-1})^{-1}\tau _i^{-2}\right )^4\right ) \le \left (2^{10}(1-\gamma _0^{-1})^{-1}\tau _i^{-2}\right )^4 . \end{aligned}$$

Since $\tau _i=\tau _0^{2^i}\gamma _0^{-2^i+1}$ by (11), it follows that

$$\begin{aligned} d_{\ell }\le 2^k \left (2^{10}\right (1-\gamma _0^{-1}\left )^{-1}\right )^{20\ell } \left (\frac{\gamma _0}{\tau _0}\right )^{20(2^{\ell +1}-1)} . \end{aligned}$$

Repeatedly taking two copies of the output as necessary, for all $2^K$ with $2^K\ge d_{\ell }$ we obtain a correlation amplifier with parameters $(2^k,2^K,2^{\ell },\tau _0,\gamma _0)$. This completes the proof of Lemma 3. $\square$

### Copy-and-Truncate Preprocessing of the Input Dimension

We still want to remove the assumption from Lemma 3 that the input dimension is a positive integer power of 2. The following copy-and-truncate preprocessing will be sufficient towards this end.

Let $x\in \{-1,1\}^d$ and let *k* be a positive integer. Define the vector ${\hat{x}}\in \{-1,1\}^{2^k}$ by concatenating $\lceil 2^k/d\rceil$ copies of *x* one after another, and truncating the result to the $2^k$ first coordinates to obtain ${\hat{x}}$.

Let us study how the map $x\mapsto {\hat{x}}$ operates on a pair of vectors $x,y\in \{-1,1\}^d$. For notational compactness, let us work with relative inner products $\nu ,{\hat{\nu }}$ with $\langle x,y\rangle =\nu d$ and $\langle {\hat{x}},{\hat{y}}\rangle ={\hat{\nu }} 2^k$.

#### **Lemma 9**

*For any* $0<\tau _0<1$, $\gamma _0>1$, *and* $2^k\ge 2d\tau _0^{-1}(1-\gamma _0^{-1})^{-1}$ *we have that*

1. $$|\nu |<\tau _0$$ *implies* $$|{\hat{\nu }}|\le \gamma _0\tau _0$$,
2. $$\left| \nu \right| \ge \tau _{0}$$ *implies* $$\gamma _0^{-1}\nu \le \left| {\hat{\nu }}\right| \le \gamma _0\nu$$.

#### *Proof*

Let $\ell$ and *t* be the unique integers such that $2^k+\ell =td$ with $0\le \ell <d$. Since we are leaving out $\ell$ coordinates, we have

$$\begin{aligned} 2^{-k}(\nu td-\ell )\le {\hat{\nu }}\le 2^{-k}(\nu td+\ell ). \end{aligned}$$

Suppose that $|\nu |<\tau _0$. We have

$$\begin{aligned} |{\hat{\nu }}| \le 2^{-k}\left (|\nu |td+\ell \right ) \le 2^{-k}\left (|\nu |2^k+2\ell \right ) \le \tau _0+2^{1-k}d. \end{aligned}$$

Observe that $1-\gamma _0^{-1}\le \gamma _0-1$. Since by hypothesis

$$\begin{aligned} 2^k\ge 2d\tau _0^{-1}(1-\gamma _0^{-1})^{-1}\ge 2d\tau _0^{-1}(\gamma _0-1)^{-1}, \end{aligned}$$

we thus have $|{\hat{\nu }}|\le \gamma _0\tau _0$.

For $\nu \ge \tau _0$ we have

$$\begin{aligned} \nu -2^{-k}d\le 2^{-k}(\nu 2^k-d)\le {\hat{\nu }}\le 2^{-k}(\nu 2^k+2d)\le \nu +2^{1-k}d. \end{aligned}$$

Similarly, for $\nu \le -\tau _0$ we have

$$\begin{aligned} \nu -2^{1-k}d\le 2^{-k}(\nu 2^k-2d)\le {\hat{\nu }}\le 2^{-k}(\nu 2^k+d)\le \nu +2^{-k}d. \end{aligned}$$

By hypothesis we have

$$\begin{aligned} 2^k\ge 2d\tau _0^{-1}\max \left ((1-\gamma _0^{-1})^{-1},(\gamma _0-1)^{-1}\right ). \end{aligned}$$

Thus we have both $\gamma _0^{-1}\nu \le {\hat{\nu }}\le \gamma _0\nu$ if $\nu \ge \tau _0$, and $\gamma _0\nu \le {\hat{\nu }}\le \gamma _0^{-1}\nu$ if $\nu \le -\tau _0$. $\square$

### Completing the Proof of Theorem 1

Let $d,K,\ell ,\tau ,\gamma$ be parameters meeting the constraints in Theorem 1, in particular the constraint (4). To construct a required amplifier *f*, we preprocess each input vector *x* with copy-and-truncate, obtaining a vector ${\hat{x}}$ of length $2^{k}$. We then then apply an amplifier ${\hat{f}}:\{-1,1\}^{2^k}\rightarrow \{-1,1\}^{2^K}$ given by Lemma 3. In symbols, we define $f:\{-1,1\}^{d}\rightarrow \{-1,1\}^{2^K}$ for all $x\in \{-1,1\}^d$ by $f(x)={\hat{f}}({\hat{x}})$. It is immediate from Lemmas 3 and 9 that the resulting composition is explicit.

We begin by relating the given parameters of Theorem 1 to those of Lemma 3. Take $\gamma _0=\gamma ^{1/2}$, $\tau _0=\tau \gamma ^{-1}$, and select the minimal value of *k* so that the constraint in Lemma 9 is satisfied; that is $2^{k}$ is constrained as follows,

$$\begin{aligned} 2d (1-\gamma ^{-1/2})^{-1} \gamma \tau ^{-1} \le 2^{k} < 4d (1-\gamma ^{-1/2})^{-1} \gamma \tau ^{-1}. \end{aligned}$$

Substituting this upper bound into the bound of Lemma 3, we get a lower bound for $2^{K}$,

16 $$\begin{aligned} 2^{K} \ge 2^{-8} d \left( 2^{10} (1- \gamma ^{-1/2})^{-1} \right) ^{20 \ell + 1} \frac{\gamma }{\tau }\left( \frac{\gamma ^{60}}{\tau ^{40}}\right) ^{2^{\ell }}\frac{\tau ^{20}}{\gamma ^{30}} . \end{aligned}$$

Observe that an integer $2^K$ satisfying (4) also satisfies (16). We have not attempted to optimise our construction, and prefer the the statement of Theorem 1 as it is reasonably clean and is sufficient to prove Theorem 2.

Let us study how the map $x\mapsto f(x)$ operates on a pair of vectors $x,y\in \{-1,1\}^d$. For notational compactness, again we work with relative inner products $\nu ,{\hat{\nu }},\phi$ with $\langle x,y\rangle =\nu d$, $\langle {\hat{x}},{\hat{y}}\rangle ={\hat{\nu }} 2^k$, and $\langle f(x),f(y)\rangle =\phi 2^K$. Observe that in the notation of the proof of Lemma 3, we have ${\hat{\nu }}=\nu _0$ and $\phi =\nu _\ell$.

#### **Lemma 10**

*If* $$\left| \nu \right| < \tau$$ *then* $$\left| \phi \right| \le (\gamma \tau )^{2^{\ell }}$$.

#### *Proof*

First we show that $\left| {\hat{\nu }}\right| \le \gamma _{0} \tau$, dividing into cases as in Lemma 9. If $\left| \nu \right| < \tau _{0}$ then $\left| {\hat{\nu }} \right| < \gamma _{0}\tau _{0} = \gamma _{0}^{-1} \tau \le \gamma _{0} \tau$. If $\tau _{0} \le \nu < \tau$ then ${\hat{\nu }} \le \gamma _{0} \nu \le \gamma _{0}\tau$. If $-\tau < \nu \le \tau _{0}$ then ${\hat{\nu }} \ge \gamma _{0} \nu \ge -\gamma _{0}\tau$.

To complete the proof, we condition on $\left| {\hat{\nu }} \right|$. If $\left| {\hat{\nu }} \right| \le \tau _{0}$ then Lemma 8 applies, and we have

$$\begin{aligned} \left| \phi \right| = \left| \nu _{\ell } \right| \le \tau _{0}^{2^{\ell }}\gamma _{0}^{2^{\ell }-1} < (\tau \gamma )^{2^{\ell }} . \end{aligned}$$

Otherwise, $\tau _{0} \le \left| {\hat{\nu }} \right| < \tau$ and by Lemma 7 we have

$$\begin{aligned} 0 < \phi = \nu _{\ell } \le \nu _{0}^{2^{\ell }} \gamma _{0}^{2^{\ell }-1} \le \tau ^{2^{\ell }} \gamma _{0}^{2^{\ell }-1} \le (\tau \gamma )^{2^{\ell }} . \end{aligned}$$

$$\square$$

#### **Lemma 11**

*If* $$\left| \nu \right| \ge \tau$$ *then* $$(\nu \gamma ^{-1})^{2^{\ell }} \le \phi \le (\nu \gamma )^{2^{\ell }}$$.

#### *Proof*

It will be convenient to split the analysis according to whether $\nu$ is positive or negative. Suppose first that $\nu \ge \tau$.

Then by Lemma 9 we have that

17 $$\begin{aligned} \gamma _{0}^{-1} \nu \le {\hat{\nu }} \le \gamma _{0} \nu . \end{aligned}$$

Since ${\hat{\nu }} \ge \nu \gamma _{0}^{-1} \ge \tau \gamma _{0}^{-1} = \tau _{0}\gamma _{0}\ge \tau _{0}$, Lemma 7 applies, yielding

$$\begin{aligned} {\hat{\nu }} \gamma _{0}^{-2^{\ell } + 1} \le \nu _{\ell } \le {\hat{\nu }}^{2^{\ell }} \gamma _{0}^{2^{\ell } -1}. \end{aligned}$$

Now, we substitute $\phi = \nu _{\ell }$ and bound ${\hat{\nu }}$ as in (17),

$$\begin{aligned} \left( \nu \gamma _{0}^{-1} \right) ^{2^{\ell }} \gamma _{0}^{-2^{\ell } + 1} \le \phi \le \left( \nu \gamma _{0}^{-1} \right) ^{2^{\ell }} \gamma _{0}^{2^{\ell } - 1}. \end{aligned}$$

Substituting $\gamma = \gamma _{0}^{1/2}$ and observing that $\gamma \ge 1$ provides the required bound

$$\begin{aligned} \left( \nu \gamma ^{-1} \right) ^{2^{\ell } } \le \phi \le \left( \nu \gamma \right) ^{2^{\ell } }. \end{aligned}$$

The case that $\nu \le -\tau$ essentially follows from multiplying all inequalities in the positive case by $-1$. $\square$

Now, *f* satisfies (2) and (3) with $p = 2^{\ell }$ by Lemmas 10 and 11 respectively. This completes the proof of Theorem 1. $\square$

## A Deterministic Algorithm for Outlier Correlations

This section proves Theorem 2. We start by describing the algorithm, then parameterize it and establish its correctness, and finally proceed to analyze the running time.

### The Algorithm

Fix the constants $\epsilon ,\tau _\mathrm {max},\delta ,C$ as in Theorem 2. Based on these constants, fix the constants $0<\sigma <1$ and $\gamma >1$. (We fix the precise values of $\sigma$ and $\gamma$ later during the analysis of the algorithm, and stress that $\sigma ,\gamma$ do not depend on the given input.)

Suppose we are given as input the parameters $0<\tau<\rho <1$ and $X,Y\subseteq \{-1,1\}^d$ with $|X|=|Y|=n$ so that the requirements in Theorem 2 hold. We work with a correlation amplifier $f:\{-1,1\}^d\rightarrow \{-1,1\}^D$ with parameters $(d,D,p,\tau ,\gamma )$. (We fix the precise values of the parameters *p* and *D* later during the analysis of the algorithm so that *f* originates from Theorem 1.)

The algorithm proceeds as follows. First, apply *f* to each vector in *X* and *Y* to obtain the sets $X_f$ and $Y_f$. Let $s=\lfloor n^{\sigma }\rfloor$. Second, partition the *n* vectors in both $X_f$ and $Y_f$ into $\lceil n/s\rceil$ buckets of size at most *s* each, and take the vector sum of the vectors in each bucket to obtain the sets ${\tilde{X}}_f,{\tilde{Y}}_f\subseteq \{-s,-s+1,\ldots ,s-1,s\}^D$ with $|{\tilde{X}}_f|,|{\tilde{Y}}_f|\le \lceil n/s\rceil$. Third, using fast rectangular matrix multiplication on ${\tilde{X}}_f$ and ${\tilde{Y}}_f$, compute the matrix *Z* whose entries are the inner products $\langle {\tilde{x}},{\tilde{y}}\rangle$ for all ${\tilde{x}}\in {\tilde{X}}_f$ and all ${\tilde{y}}\in {\tilde{Y}}_f$. Fourth, iterate over the entries of *Z*, and whenever the *detection inequality*

18 $$\begin{aligned} \langle {\tilde{x}},{\tilde{y}}\rangle >n^{2\sigma }(\tau \gamma )^p D \end{aligned}$$

holds, brute-force search for outliers among the at most $s^2$ inner products in the corresponding pair of buckets. Output any outliers found.

### Parameterization and Correctness

Let us now parameterize the algorithm and establish its correctness. Since $\gamma >1$ is a constant and assuming that *p* is large enough, by Theorem 1 we can select *D* to be the integer power of 2 with

$$\begin{aligned} \frac{1}{2}d\left (\frac{\gamma }{\tau }\right )^{Cp} < D\le d\left (\frac{\gamma }{\tau }\right )^{Cp}. \end{aligned}$$

Recall that we write $\alpha$ for the exponent of rectangular matrix multiplication. To apply fast rectangular matrix multiplication in the third step of the algorithm, we want

19 $$\begin{aligned} D\le 2\left (\frac{n}{s}\right )^\alpha , \end{aligned}$$

so recalling that $d\le n^\delta$ and $n^\sigma -1<s$, it suffices to require that

$$\begin{aligned} \left (\frac{\gamma }{\tau }\right )^{Cp} \le n^{(1-\sigma )\alpha -\delta }. \end{aligned}$$

Let us assume for the time being that $(1-\sigma )\alpha -\delta >0$. (We will justify this assumption later when we choose a value for $\sigma$.) Let *p* be the unique positive-integer power of 2 such that

20 $$\begin{aligned} \frac{((1-\sigma )\alpha -\delta )\log n}{2C\log \frac{\gamma }{\tau }}< p\le \frac{((1-\sigma )\alpha -\delta )\log n}{C\log \frac{\gamma }{\tau }}. \end{aligned}$$

We will later, when fixing $\sigma$ and $\gamma$, make sure that the right-hand side in (20) is at least 1, so that *p* exists and is positive.

Let us now consider a single entry $\langle {\tilde{x}},{\tilde{y}}\rangle$ in *Z*, and analyze how the corresponding (at most $s^2$) inner products $\langle x,y \rangle$ between the two buckets of input vectors relate to the detection inequality (18). We make two claims:

Claim 1 (background case). If all of the inner products have $|\langle x,y \rangle | \le \tau d$, then (18) *does not* hold, so the algorithm will not search inside this pair of buckets. This claim will be used to control the running time. The claim follows directly from (2) and (3), since there are at most $s^2 \le n^{2\sigma }$ inner products, each having $|\langle f(x),f(y)\rangle | \le (\tau \gamma )^p D$.

Claim 2 (outlier case). If at least one of the inner products has $|\langle x,y \rangle | \ge \rho d$, then (18) *holds*, so the algorithm searches inside this pair of buckets. This guarantees that the outliers are detected.

Note that in the third case, namely, if some inner products have $|\langle x,y \rangle | > \tau d$ but none has $|\langle x,y \rangle | \ge \rho d$, we make no claim on whether (18) holds or not. The algorithm is not required to search inside such pairs of buckets (since there are no outliers there), but may so do without hindering our overall running time bound.

We proceed to parameterize the algorithm so that Claim 2 holds. In the outlier case, by (2) and (3), there is at least one inner product with $\langle f(x),f(y) \rangle \ge (\rho \gamma ^{-1})^p D$, and the remaining at most $n^{2\sigma }$ inner products have $\langle f(x),f(y) \rangle \ge -(\tau \gamma )^p D$. Thus in the outlier case we have

21 $$\begin{aligned} \langle {\tilde{x}}, {\tilde{y}} \rangle \ge (\rho \gamma ^{-1})^pD - n^{2\sigma }(\tau \gamma )^pD. \end{aligned}$$

For Claim 2 we need the detection inequality (18) to hold whenever (21) holds. Towards this end, it suffices to require that

$$\begin{aligned} \left (\rho \gamma ^{-1}\right )^p-n^{2\sigma }\left (\tau \gamma \right )^p > n^{2\sigma }\left (\tau \gamma \right )^p. \end{aligned}$$

Rearranging and solving for *p*, we require that

22 $$\begin{aligned} p>\frac{1+2\sigma \log n}{\log \frac{\rho }{\tau \gamma ^2}}. \end{aligned}$$

From (20) and (22) we thus see that it suffices to have

$$\begin{aligned} p>\frac{((1-\sigma )\alpha -\delta )\log n}{2C\log \frac{\gamma }{\tau }} \ge \frac{1+2\sigma \log n}{\log \frac{\rho }{\tau \gamma ^2}}, \end{aligned}$$

or equivalently,

23 $$\begin{aligned} \frac{\log \frac{\rho }{\tau \gamma ^2}}{\log \frac{\gamma }{\tau }} \ge \frac{\frac{2C}{\log n}+4C\sigma }{(1-\sigma )\alpha -\delta }. \end{aligned}$$

Let us derive a lower bound for the left-hand side of (23). Fix the constant $\gamma >1$ so that $\log \gamma =-\frac{\epsilon \log \tau _\mathrm {max}}{100{,}000}$. By our assumptions we have $\tau \le \tau _\mathrm {max}$ and $1-\log _\tau \rho \ge \epsilon$, so we have the lower bound

$$\begin{aligned} \frac{\log \frac{\rho }{\tau \gamma ^2}}{\log \frac{\gamma }{\tau }} = \frac{\log \rho -\log \tau -2\log \gamma }{\log \gamma -\log \tau } = \frac{1-\log _\tau \rho +\frac{2\log \gamma }{\log \tau }}{1-\frac{\log \gamma }{\log \tau }} \ge \frac{\epsilon +\frac{2\log \gamma }{\log \tau _\mathrm {max}}}{1-\frac{\log \gamma }{\log \tau _\mathrm {max}}}> 0.99\epsilon . \end{aligned}$$

Thus, (23) holds for all large enough *n* when we require

$$\begin{aligned} 0.99\epsilon \ge \frac{4C\sigma }{(1-\sigma )\alpha -\delta }. \end{aligned}$$

Since $\alpha \epsilon <1$, we have that (23) holds when we set

$$\begin{aligned} \sigma =\frac{0.99\epsilon (\alpha -\delta )}{4C+1} \le \frac{0.99\epsilon (\alpha -\delta )}{4C+0.99\alpha \epsilon }. \end{aligned}$$

We also observe that $(1-\sigma )\alpha -\delta >0$, or equivalently, $\sigma <(\alpha -\delta )/\alpha$ holds for our choice of $\sigma$.

Having now fixed $\sigma$ and $\gamma$, we observe that in terms of assumption 2 of the statement of Theorem 2, we have $\gamma =c_1$ and $\frac{(1-\sigma )\alpha -\delta }{C}=c_2$. Thus the assumption $\tau \ge c_1 n^{-c_2}$ guarantees that the right-hand side of (20) is at least 1, which was required for the existence of *p*. This completes the parameterization of the algorithm.

### Running Time

Let us now analyze the running time of the algorithm. The first and second steps run in time ${\tilde{O}}(nD)$ since $p=O(\log n)$ by (20) and *f* originates from Theorem 1 and hence is explicit. From (19) and $n^{\sigma }-1<s$, we have $nD\le 4n^{1+(1-\sigma )\alpha }\le 4n^{2-\sigma }$. Since (19) holds, the third step of the algorithm runs in time $O\left ((n/s)^{2+\eta }\right )$ for any constant $\eta >0$ that we are free to choose. Since $n/s\le 2n^{1-\sigma }$ for all large enough *n*, we can choose $\eta >0$ so that $(2+\eta )(1-\sigma )\le 2-\sigma$. Thus, the first, second, and third steps together run in time $O(n^{2-\sigma })$. The fourth step runs in time $O(n^{2-\sigma }+qs^2d)$. Indeed, observe from Claim 1 in § 4.2 that the detection inequality (18) holds for at most *q* entries in *Z*. We have $qs^2d\le qn^{2\sigma +\delta }$, which completes the running time analysis and the proof of Theorem 2. $\square$

## Applications

This section proves Corollaries 1 and 2.

### The Light Bulb Problem

A useful variant of the Problem 1 asks for all outlier pairs of distinct vectors drawn from a *single* set $S\subseteq \{-1,1\}^d$ rather than two sets *X*, *Y*. We observe that the single-set variant reduces to $\lceil \log |S|\rceil$ instances of the two-set variant by numbering the vectors in *S* with binary numbers from 0 to $|S|-1$ and splitting *S* into two sets $X_i,Y_i$ based on the value of the $i^\mathrm {th}$ bit for each $i=0,1,\ldots ,\lceil \log |S|\rceil -1$.

#### *Proof of Corollary 1*

We reduce to (the single-set version of) Problem 1 and apply Theorem 2. Towards this end, in Theorem 2 set $\epsilon = 1-1/\kappa$ and $\tau _{\mathrm {max}} = \rho _{\mathrm {max}}^\kappa$. Suppose we are given an instance of Problem 2 whose parameters $n,d,\rho$ satisfy the constraints. Set $\tau = \rho ^\kappa$. We observe that the constraints in Theorem 2 are satisfied since (1) $d\le n^\delta$ holds by assumption, (2) $\tau \le \tau _{\max }$ holds since $\tau = \rho ^\kappa \le \rho _{\max }^\kappa$, (3) the constants $c_1$ and $c_2$ here match those in Theorem 2, and the constraint $c_1n^{-c_2/\kappa } \le \rho$ implies $c_1n^{-c_2} \le \tau$, and (iv) $\log _\tau \rho = \frac{\log \rho }{\log \tau } = \frac{\log \rho }{\log \rho ^\kappa } = 1/\kappa \le 1-\epsilon$.

We claim that $q=1$ in almost all instances of Problem 2 whose parameters satisfy the constraints in Corollary 1. Indeed, by the Hoeffding bound (7) and the union bound, the probability that some other pair than the planted pair in an instance has inner product that exceeds $\tau d$ in absolute value is at most

$$\begin{aligned} 2n^2\exp \left (-\tau ^2d/2\right ) \le 2n^2\exp \left (-\rho ^{2\kappa }\cdot 5\rho ^{-2\kappa }\log n\right ) = 2n^{-1/2}, \end{aligned}$$

so $q=1$ with high probability as *n* increases. The claimed running time follows by substituting the chosen constants and $q=1$ to (5). $\square$

### Learning Parities with Noise

We now generalize the result for parity functions of larger constant weight, and prove Corollary 2.

#### *Proof of Corollary 2*

Fix the constants $0< \delta < \alpha$, $C>60$, $\xi >1$, $0<\theta < 1$. We will fix the value of the constant $k_0$ later. Let $k\ge k_0$ be a constant. The algorithm first draws *d* examples from a given instance of Problem 3 and then transforms these to two collections of vectors that we feed to the algorithm of Theorem 2 and then proceed to mimic the proof of Corollary 1.

Let us first set up some notation. For $A,B\subseteq [v]$, let $A\bigtriangleup B = (A{\setminus } B) \cup (B{\setminus } A)$ denote the symmetric difference of *A* and *B*. Let $x = (x(1),x(2),\ldots , x(v))\in \{-1,1\}^v$ be a Boolean *n*-vector. Let $x^A = \prod _{\ell \in A} x(\ell )$ be the product of elements indexed by *A*, with $x^\emptyset = 1$. Observe that $x^Ax^B = \prod _{i\in A}x(i)\prod _{j\in B} x(j) = \prod _{\ell \in A\bigtriangleup B} x(\ell ) = x^{A\bigtriangleup B}$. Let us write $$\left( {\begin{array}{c}[n]\\ v\end{array}}\right)$$ for the set of all *k*-subsets of [*v*].

Suppose we are now given as input an instance of Problem 3 with noise level $\eta$ that satisfies $|1-2\eta |\le \theta <1$. Furthermore, we assume that $\eta$ is part of the input. (If this is not the case, at the cost of increasing time complexity, we can search for $\eta$ using a geometric progression with limit 1/2.) With the objective of eventually applying Theorem 2, set

24 $$\begin{aligned} \rho = |1-2\eta |^\xi \end{aligned}$$

and

25 $$\begin{aligned} \tau = \rho ^\xi = |1-2\eta |^{\xi ^2}. \end{aligned}$$

In particular, we have $\tau <\rho$ since $0<|1-2\eta |<1$ and $\xi >1$. Let *d* be the least positive integer that satisfies

26 $$\begin{aligned} d \ge (2k+1+4k\zeta )\tau ^{-2}(|1-2\eta |-\rho )^{-2}\log v\, , \end{aligned}$$

where $0< \zeta < 1/2$ is constant whose value we will fix later. Draw from the given instance *d* example–label pairs $(x_i,y_i) \in \{-1,1\}^v \times \{-1,1\}$ with $i=1,2,\ldots ,d$. We use these examples to define two collections $X,Y\subseteq \{-1,1\}^d$ of vectors of sizes $$\left( {\begin{array}{c}v\\ \lfloor k/2 \rfloor \end{array}}\right)$$ and $$\left( {\begin{array}{c}v\\ \lceil k/2 \rceil \end{array}}\right)$$, respectively. For all $k\ge \lceil 1/(2\zeta )\rceil$ and all $v\ge 2k$ it is immediate that we have

$$\begin{aligned} \left( {\begin{array}{c}v\\ \lfloor k/2 \rfloor \end{array}}\right) \le \left( {\begin{array}{c}v\\ \lceil k/2 \rceil \end{array}}\right) \le v^{k(1/2+\zeta )}. \end{aligned}$$

In particular, we can assume that $|X|,|Y|\le n$ for $n=\lfloor v^{k(1/2+\zeta )}\rfloor$.

The set *X* consists of all the vectors

$$\begin{aligned} a^{J_1} = (a_1^{J_1},a_2^{J_1},\ldots ,a_d^{J_1}) \in \{-1,1\}^d \end{aligned}$$

with $a_i^{J_1} = x_i^{J_1}$ for all $i=1,2,\ldots ,d$ and $$J_1\in \left( {\begin{array}{c}[v]\\ \lfloor k/2 \rfloor \end{array}}\right)$$. The set *Y* consists of all the vectors

$$\begin{aligned} b^{J_2} = (b_1^{J_2},b_2^{J_2},\ldots ,b_d^{J_2}) \end{aligned}$$

with $b_i^{J_1} = x_i^{J_2}y_i$ for all $i=1,2,\ldots ,d$ and $$J_2\in \left( {\begin{array}{c}[v]\\ \lceil k/2 \rceil \end{array}}\right)$$.

Let us now study the distribution of inner products between vectors in *X* and *Y*. We write ${{\,\mathrm{Bin}\,}}_{\pm 1}(d,\beta )$ for a random variable that is the sum of *d* independent random variables, each of which takes the value $-1$ with probability $\beta$, and the value 1 otherwise. Observe that the expectation of ${{\,\mathrm{Bin}\,}}_{\pm 1}(d,\beta )$ is $(1-2\beta )d$.

Let $S\subseteq [v]$ with $|S|=k$ be the support of the parity function that is unknown to us. Recall that $y_i = z_ix_i^S$ with $z_i\in \{-1,1\}$ getting value $-1$ with probability $\eta$. For all $$J_1 \in \left( {\begin{array}{c}[v]\\ \lfloor k/2 \rfloor \end{array}}\right)$$ and $$J_2 \in \left( {\begin{array}{c}[v]\\ \lceil k/2 \rceil \end{array}}\right)$$ we have

$$\begin{aligned} \langle a^{J_1} , b^{J_2} \rangle = \sum _{i=1}^d x_i^{J_1}x_i^{J_2} y_i = \sum _{i=1}^d x_i^{J_1\bigtriangleup J_2} x_i^S z_i = \sum _{i=1}^d x_i^{J_1\bigtriangleup J_2 \bigtriangleup S} z_i . \end{aligned}$$

Now observe that there are two distinct cases: If $J_1\bigtriangleup J_2 \ne S$, then

27 $$\begin{aligned} \langle a^{J_1} , b^{J_2} \rangle \sim {{\,\mathrm{Bin}\,}}_{\pm 1} (d,1/2) . \end{aligned}$$

If $$J_1\bigtriangleup J_2 = S$$, then

28 $$\begin{aligned} \langle a^{J_1} , b^{J_2} \rangle = \sum _{i=1}^d x_i^{J_1\bigtriangleup J_2 \bigtriangleup S} z_i = \sum _{i=1}^d z_i \sim {{\,\mathrm{Bin}\,}}_{\pm 1}(d,\eta ). \end{aligned}$$

Hence, our task of finding the support *S* reduces to that of locating the inner products with distribution ${{\,\mathrm{Bin}\,}}_{\pm 1}(d,\eta )$ from among those with ${{\,\mathrm{Bin}\,}}_{\pm 1}(d,1/2)$.

We now argue that our choices (24), (25), and (26) suffice for the algorithm in Theorem 2 to distinguish between the two cases (27) and (28) for almost all draws of the *d* examples. Here we stress that the algorithm is deterministic, the randomness is over the draw of the examples.

From the perspective of the algorithm in Theorem 2, it suffices that (a) no pair with (27) exceeds $\tau d$ in absolute-value inner product, and (b) at least one of the at most $k^k=O(1)$ pairs with (28) has absolute-value inner product at least $\rho d$.

To control (a), from (7) we observe that

$$\begin{aligned} \Pr \left (|{{\,\mathrm{Bin}\,}}_{\pm 1}(d,1/2)| \ge \tau d\right )&\le 2 \exp \left( -\frac{\tau ^2d}{2}\right) \\&\le 2 \exp \left( -\frac{(2k+1+4k\zeta )\log v}{2(|1-2\eta |-\rho )^2} \right) \\&= 2 v^{-(2k+1+4k\zeta )(|1-2\eta |-\rho )^{-2}/2} . \end{aligned}$$

Since there are at most $n^2 \le (v^{k(1/2+\zeta )})^2 = v^{k+2\zeta }$ such pairs, we observe by the union bound that (a) holds with high probability as *v* increases since

29 $$\begin{aligned} n^2 \cdot 2 v^{-(2k+1+4k\zeta )(|1-2\eta |-\rho )^{-2}/2} \le 2 v^{-(1/2)(|1-2\eta |-\rho )^{-2}} . \end{aligned}$$

To control (b), select any fixed pair with (28). From (7) we have

30 $$\begin{aligned} \begin{aligned}&\Pr \left (|{{\,\mathrm{Bin}\,}}_{\pm 1}(d,\eta )-(1-2\eta )d| \ge (|1-2\eta |-\rho ) d\right ) \\&\quad \le \; 2\exp \left( -\frac{(|1-2\eta |-\rho )^2d}{2}\right) \\&\quad \le \; 2\exp \left( -\frac{(2k+1+4k\zeta )\log v}{2\tau ^2} \right) \\&\quad = \; 2 v^{-\frac{2k+1+4k\zeta }{2\tau ^2}}. \end{aligned} \end{aligned}$$

Thus, (b) holds with high probability as *v* increases.

It remains to verify the constraints for the parameters $n,d,\rho ,\tau$ in Theorem 2. Suppressing the constants, our choice of *d* in (26) is ${\varTheta }(k)\cdot |1-2\eta |^{-{\varTheta }(1)}\cdot \log v$. For Theorem 2 to apply, this must be bounded from above by $n^\delta = v^{{\varTheta }(k)}$, which holds if $|1-2\eta |\ge v^{-{\varTheta }(k)}$. This holds by assumption for sufficiently large *k*. Select $k_0$ so that this constraint holds and $k_0\ge \lceil 1/(2\zeta )\rceil$. We can choose $\tau _{\max } = \theta$ and $\epsilon = 1-1/\xi$. We then have $\tau = |1-2\eta |^{\xi ^2}< \tau _{\max } < 1$ by assumption, as required. Since $n \ge v^{k/2}$, we also have by assumption

$$\begin{aligned} \tau = |1-2\eta |^{\xi ^2} \ge {c_1}^{\xi ^2} v^{-c_2 k/2} \ge c_1 n^{-c_2} \end{aligned}$$

as required. The constants $c_1$ and $c_2$ here match those in Theorem 2. Furthermore by the choice of $\epsilon$ we have

$$\begin{aligned} \frac{\log \rho }{\log \tau } = \frac{\log \rho }{\log \rho ^\xi } = 1/\xi = 1-\epsilon \, , \end{aligned}$$

as required. So the constraints of Theorem 2 are satisfied. For brevity, let $E = \frac{0.99\epsilon (\alpha -\delta )}{4C+1}$ and take $\zeta =E/4$. Thus, we have

31 $$\begin{aligned} n^{2-E} \le \left( v^{k(1/2+\zeta )}\right) ^{2-E} \le v^{k(1-0.245025(\alpha -\delta )^2(1-1/\xi )^2(1+4C)^{-2})}. \end{aligned}$$

The claimed running time (6) follows by observing that (31) subsumes the time it takes to construct the collections *X* and *Y* together with the time it takes to search the *q* pairs of buckets with $q\le k^k=O(1)$ inside the algorithm of Theorem 2.

Inserting our choices (24) and (25) into (26) and approximating upwards with $\zeta \le 1$ and $|1-2\eta |^{2\xi ^2+2} (1-\theta ^{\xi -1})^2 \le \tau ^2(|1-2\eta |-\rho )^2$ yields

$$\begin{aligned} d \ge \frac{6k}{|1-2\eta |^{2(\xi ^2+1)}(1-\theta ^{\xi -1})^2}\log v. \end{aligned}$$

$$\square$$

## Nonconstructive Existence and a Lower Bound

This section shows that nontrivial correlation amplifiers exist and establishes a lower bound on the output dimension *D* of any correlation amplifier. The former is done by a routine application of the Hoeffding bound and the latter by applying results of Alon [4].

### Low-Dimensional Amplifiers Exist

By combining the Hoeffding bound with the union bound, we observe that low-dimensional amplifiers exist.

#### **Lemma 12**

(Existence) *There exists a correlation amplifier* $f: \{-1, 1\}^{d} \rightarrow \{-1, 1\}^{D}$ *with parameters* $(d, D, p, \tau , \gamma )$ *whenever* $0< \tau < 1$, $\gamma > 1$, *and* *d*, *p*, *D* *are positive integers satisfying*

32 $$\begin{aligned} D\ge 3d\left( \gamma ^{p} - 1\right) ^{-2}\left( \frac{\gamma }{\tau }\right) ^{2p} . \end{aligned}$$

#### *Proof*

Let $f: \{-1,1\}^{d} \rightarrow \{-1, 1\}^{D}$ be the function which maps *x* onto *D* entries of $x^{\otimes p}$ chosen independently at random. That is, each entry of the vector *f*(*x*) is the product of *p* entries of *x*, chosen independently and uniformly at random.

Let $x, y \in \{-1, 1\}^{d}$ be a fixed pair of vectors, set $c = D (1-\gamma ^{-p})\tau ^{p}$, and suppose that the following inequality holds,

33 $$\begin{aligned} \left| \langle f(x), f(y) \rangle - D \left( \frac{\langle x, y \rangle }{d}\right) ^{p} \right| \le c . \end{aligned}$$

Observe that if $\left| \langle x, y\rangle \right| < \tau d$ then (33) implies

$$\begin{aligned} \left| \langle f(x), f(y) \rangle \right|&\le D \left( \frac{\langle x, y \rangle }{d}\right) ^{p} + D (1-\gamma ^{-p})\tau ^{p} \\&\le D \tau ^{p} + D (1-\gamma ^{-p})\tau ^{p} \\&\le (\tau \gamma )^{p} D . \end{aligned}$$

The final inequality holds because $2 - \gamma ^{-p} \le \gamma ^{p}$ is logically equivalent to $(\gamma ^{p} - 1)^{2} \ge 0$. Similarly, if $\left| \langle x, y\rangle \right| \ge \tau d$ then (33) implies the following upper bound,

$$\begin{aligned} \langle f(x), f(y) \rangle&\le D \left( \frac{\langle x, y \rangle }{d}\right) ^{p} + D (1-\gamma ^{-p})\tau ^{p} \\&\le D \left( \frac{\langle x, y \rangle }{d}\right) ^{p} + D (1-\gamma ^{-p})\left( \frac{\langle x, y \rangle }{d}\right) ^{p}\\&\le \left( \frac{\gamma \langle x, y \rangle }{d}\right) ^{p}D. \end{aligned}$$

We also obtain a lower bound from (33) when $\left| \langle x, y\rangle \right| \ge \tau d$,

$$\begin{aligned} \langle f(x), f(y) \rangle&\ge D \left( \frac{\langle x, y \rangle }{d}\right) ^{p} - D (1-\gamma ^{-p})\tau ^{p} \\&\ge D \left( \frac{\langle x, y \rangle }{d}\right) ^{p} - D (1-\gamma ^{-p})\left( \frac{\langle x, y \rangle }{d}\right) ^{p}\\&\ge \left( \frac{\langle x, y \rangle }{\gamma d}\right) ^{p}D. \end{aligned}$$

In fact, (33) implies conditions (2) and (3) in Definition 1. So if the function *f* satisfies (33) for all $x, y \in \{-1, 1\}^{d}$, then *f* is a correlation amplifier. We use Theorem 3 to bound the probability that (33) fails, and take a union bound over the range of *f* to establish a non-constructive existence result for sufficiently large *D*.

Define the random variable $Z_{f} = \langle f(x), f(y)\rangle$. Since *f*(*x*) is a restriction onto *D* entries of $x^{\otimes p}$ chosen uniformly at random, we have

$$\begin{aligned} E[Z_{f}] = D \left( \frac{\langle x, y\rangle }{d}\right) ^{p}. \end{aligned}$$

Observe that $Z_{f} = \sum _{i=1}^{D} Z_{f,i}$ where $Z_{f,i}$ is the product of the $i^{\text {th}}$ entries of *f*(*x*) and *f*(*y*). In particular, $-1 \le Z_{f,i} \le 1$ holds for $i = 1, 2, \ldots , D$. Summing over the $Z_{f,i}$ in (7), the probability that (33) fails to hold is bounded above by

$$\begin{aligned} \text {Pr} \left( Z_{f} - {\mathrm {E}}[Z_{f}] \ge c \right) \le e^{-\frac{c^2}{2D} } . \end{aligned}$$

Taking a union bound over all $x, y \in \{-1, 1\}^{d}$, there exists a correlation amplifier with parameters $(d, D, p, \tau , \gamma )$ whenever

$$\begin{aligned} 2^{2d} e^{-\frac{c^2}{2D}} < 1. \end{aligned}$$

Solving for *D*, we get

$$\begin{aligned} D\ge \frac{d\ln 16}{\tau ^{2p}\left( 1- \gamma ^{-p}\right) ^2} . \end{aligned}$$

Simplifying this expression and approximating $\ln 16$ by 3 completes the proof. $\square$

### Lower Bound on Output Dimension

We next show a lower bound on the output dimension *D* of any correlation amplifier, when the other parameters *d*, *p*, $\tau$ and $\gamma$ are given. The proof is based on taking a collection of *N* vectors $x_i \in \{-1,1\}^d$, with all pairs below the background threshold $\tau$, and then bounding the number of their images $f(x_i) \in \{-1,1\}^D$, whose absolute pairwise correlations are required to be below $\epsilon = (\tau \gamma )^p$ by Definition 1.

#### **Lemma 13**

*There is a collection of* $N=\exp (\tau ^2 d/4)$ *vectors* $x_1,x_2,\ldots ,x_N \, \in \{-1,1\}^d$ *such that* $|\langle x_i, x_j \rangle |< \tau d$ *for all* $i \ne j$.

#### *Proof*

We show this by the probabilistic argument. We call a pair of vectors bad if $|\langle x_i, x_j \rangle | \ge \tau d$. Let a collection of vectors $X_1,X_2,\ldots ,X_N$ be chosen uniformly at random from $\{-1,1\}^d$. Consider a pair $X_i,X_j$ with $i \ne j$, and let $Z_{ij} = \langle X_i,X_j \rangle$. Now $Z_{ij}$ is a sum of *d* independent random variables in $[-1, 1]$, with $\text {E}[Z_{ij}] = 0$. Applying the two-sided Hoeffding bound with $c=\tau d$, we observe that the pair $X_i,X_j$ is bad with probability

$$\begin{aligned} \text {Pr}(|\langle X_i,X_j \rangle | \ge \tau d) = \text {Pr}(|Z_{ij} - \text {E}[Z_{ij}]| \ge \tau d) \le 2 \exp (-\tau ^2 d/2). \end{aligned}$$

Since there are less than $N^2/2 = (1/2)\exp (\tau ^2 d/2)$ pairs of vectors, the expected number of bad pairs is less than 1. Thus in at least one collection there are no bad pairs. $\square$

To bound the number of the image vectors, we use a combinatorial result from Alon [4] to bound the rank of their correlation matrix. We will require the following lemmas.

#### **Lemma 14**

(Alon, Lemma 9.1 [4]) *Let* $A = (a_{ij})$ *be an* $N \times N$ *real, symmetric matrix with* $a_{ii}=1$ *and* $|a_{ij}| \le \epsilon$ *for all* $i \ne j$. *Then* ${{\,\mathrm{rank}\,}}(A) \ge \frac{N}{1 + (N-1)\epsilon ^{2}}$. *In particular, if* $\epsilon \le \frac{1}{\sqrt{N}}$, *then* ${{\,\mathrm{rank}\,}}(A) \ge \frac{N}{2}$.

#### **Lemma 15**

(Alon, Lemma 9.2 [4]) *Let* $B=(b_{ij})$ *be an* $N \times N$ *matrix with* ${{\,\mathrm{rank}\,}}(B)=D'$, *and let* $A = (b_{ij}^k)$, *where* *k* *is a positive integer. Then* $${{\,\mathrm{rank}\,}}(A) \le \left( {\begin{array}{c}D'+k-1\\ k\end{array}}\right)$$.

The next lemma is in essence Alon’s Theorem 9.3 [4], modified to avoid any asymptotic notation. All logarithms here are in base 2.

#### **Lemma 16**

*Let* $B=(b_{ij})$ *be an* $N \times N$ *real, symmetric matrix with* $b_{ii}=1$ *and* $|b_{ij}| \le \epsilon$ *for all* $i \ne j$, *where* $1/\sqrt{N} \le \epsilon \le 1/100$, *and* ${{\,\mathrm{rank}\,}}(B)=D'$. *Then*

34 $$\begin{aligned} D' \ge \left (\frac{r}{5}\right ) \left (\frac{1}{\epsilon }\right )^{2r/(r+1)} \end{aligned}$$

*where* $$r = (\log N)/(2 \log (1/\epsilon ))$$.

#### *Proof*

Choose *r* as stated. Note that by the assumed range of $\epsilon$, we have $r \ge 1$. Let further $k = \lceil r \rceil$, so in particular $1 \le r \le k < r+1$.

Let $A = (a_{ij}) = (b_{ij}^k)$. Since the off-diagonal elements of *B* satisfy $|b_{ij}|<\epsilon$, it follows from the choice of *k* that the off-diagonal elements of *A* satisfy $|a_{ij}| \le \epsilon ^k \le \epsilon ^r = 1/\sqrt{N}$. Combining Lemmas 14 and 15, we have

$$\begin{aligned} N/2 \le {{\,\mathrm{rank}\,}}(A) \le \left( {\begin{array}{c}D'+k-1\\ k\end{array}}\right) \le \left( \frac{e(D'+k-1)}{k}\right) ^k \le \left( \frac{e(D'+r)}{r}\right) ^{r+1}. \end{aligned}$$

Taking logarithms and rearranging the inequality we obtain

$$\begin{aligned} \log \left( 1+\frac{D'}{r}\right) \ge \frac{\log (N/2)}{r+1} - \log e \ge \frac{\log N}{r+1} - 2, \end{aligned}$$

implying

$$\begin{aligned} 1+\frac{D'}{r} \ge \frac{2^{(\log N) / (r+1)}}{4}. \end{aligned}$$

Observing that $\log N = r \log (1/\epsilon ^2)$, we get

$$\begin{aligned} 1+\frac{D'}{r} \ge \frac{1}{4} \left (\frac{1}{\epsilon }\right )^{2r/(r+1)} \end{aligned}$$

and, since $\epsilon \le 1/100$ and $r\ge 1$, this implies

$$\begin{aligned} D' \ge \left (\frac{r}{5}\right ) \left (\frac{1}{\epsilon }\right )^{2r/(r+1)} \end{aligned}$$

as stated. $$\square$$

#### *Remark*

The parameter *r* measures, in a sense, the distance from the case of an extremely low correlation requirement $\epsilon =1/\sqrt{N}$. If *r* tends to infinity, the exponent $2r/(r+1)$ approaches 2, matching the asymptotic form given by Alon [4]. However, with small *r* the exponent diminishes, reaching 1 in the limiting case $r=1$, that is, when $\epsilon =1/\sqrt{N}$. In the limiting case a direct application of Lemma 14 would give the better linear bound $D' \ge N/2$.

We can now combine Lemmas 13 and 16 to get a lower bound on output dimension.

#### **Lemma 17**

(Lower bound on output dimension) *The output dimension of a correlation amplifier with parameters* $(d,D,p,\tau ,\gamma )$ *is bounded by*

$$\begin{aligned} D \ge \frac{1}{5} \left (\frac{1}{\gamma \tau }\right )^p \end{aligned}$$

*when* $$(\gamma \tau )^p \le 1/100$$ *and* $$p \le \frac{(\log e)\tau ^2d}{8\log (\frac{1}{\gamma \tau })}$$.

#### *Proof*

By Lemma 13 there is a collection of $N=\exp (\tau ^2 d/4)$ vectors $x_1,x_2,\ldots , x_N \in \{-1,1\}^d$ with correlations below $\tau$ in absolute value. By Definition 1 their images $u_i = f(x_i) \in \{-1,1\}^D$ have correlations below $\epsilon = (\gamma \tau )^p$ in absolute value.

Consider the $N \times N$ correlation matrix $B = (b_{ij}) = (\langle u_i, u_j \rangle /D)$. It is real and symmetric, with diagonal elements $b_{ii}=1$ and off-diagonals satisfying $|b_{ij}| \le \epsilon$. We observe that $D' = {{\,\mathrm{rank}\,}}(B) \le D$. Applying Lemma 16 we have

$$\begin{aligned} r = \frac{\log N}{2 \log (1/\epsilon )} = \frac{(\log e) \tau ^2 d}{8 p \log (\frac{1}{\gamma \tau })} \ge 1, \end{aligned}$$

and

35 $$\begin{aligned} D \ge D' \ge \left (\frac{r}{5}\right ) \left (\frac{1}{\epsilon }\right )^{2r/(r+1)} \ge \frac{1}{5} \left (\frac{1}{\gamma \tau }\right )^p \end{aligned}$$

as claimed. $$\square$$

#### *Remark*

At the limiting case where $p = \frac{(\log e)\tau ^2d}{8\log (\frac{1}{\gamma \tau })}$, we have $r=1$ and $\epsilon = 1/\sqrt{N} = \exp (-t^2 d / 8)$, and the bound (35) becomes $D \ge \exp (\tau ^2 d/8)$. For *p* greater than the limit, one can essentially map all of the $N=\exp (\tau ^2 d/4)$ input vectors to *orthogonal* output vectors of dimension $D \le 2N$ using a Hadamard matrix, in which case (2) holds for arbitrary $p>1$.

## Appendix: An Expander Family

This section proves Lemma 2 following Reingold et al. [34]; we present the proof for completeness of exposition only with no claim of originality. Following Reingold et al. [34] we will work with *normalized* eigenvalues. To avoid confusion with the unnormalized treatment in the manuscript proper, we say that a graph is a $[D,{\varDelta },\lambda ]$-*graph* if the graph has *D* vertices, is ${\varDelta }$-regular, and $|\lambda _2|/{\varDelta }\le \lambda$. (Here $|\lambda _2|$ is the unnormalized second eigenvalue as defined in the manuscript proper.)

We refer to Sections 2.3 and 3.1 of Reingold et al. [34] for the definition of the square $G^2$ of a graph *G*, the tensor product $G_1\otimes G_2$ of graphs $G_1,G_2$, and the zigzag product $G\textcircled {Z}H$ of graphs *G*, *H*. The following omnibus result collects elements of Proposition 2.3, Proposition 2.4, Theorem 3.2 and Theorem 4.3 of [34] which will be sufficient to control the second normalized eigenvalue for our present purposes. (We choose to omit the details of the rotation maps with the understanding that they can be found in [34].)

### **Lemma 18**

(Reingold et al. [34]) *The following bounds hold.*

1. *If* *G* *is a* $[D,{\varDelta },\lambda ]$-*graph, then* $G^{2}$ *is a* $[D,{\varDelta }^{2},\lambda ^{2}]$-*graph.*
2. *If* $G_1$ *is a* $[D_1,{\varDelta }_1,\lambda _1]$-*graph and* $G_2$ *is a* $[ D_2,{\varDelta }_2,\lambda _2]$-*graph,*
  *then* $G_1\otimes G_2$ *is a* $[D_1D_2,{\varDelta }_1{\varDelta }_2,\max (\lambda _1,\lambda _2)]$-*graph.*
3. *If* *G* *is a* $[D_1,{\varDelta }_1,\lambda _1]$-*graph and* *H* *a* $[{\varDelta }_1,{\varDelta }_2,\lambda _2]$-*graph,*
  *then* $G \textcircled {Z}H$ *is a* $[D_1{\varDelta }_1,{\varDelta }_2^2,f(\lambda _1,\lambda _2)]$-*graph with*
  $$\begin{aligned} f(\lambda _1,\lambda _2) = \frac{1}{2}\left( 1-\lambda _2^2\right) \lambda _1 + \frac{1}{2}\sqrt{\left( 1-\lambda _2^2\right) ^2\lambda _1^2 + 4\lambda _2^2} \le \lambda _1 + \lambda _2. \end{aligned}$$

Let us study the following sequence of graphs. Let *H* be a $[D,{\varDelta },\lambda ]$-graph. Let $G_1=H^2$, $G_2 = H\otimes H$, and for $t=3,4,\ldots$ let

36 $$\begin{aligned} G_t = \left( G_{\left\lceil \frac{t-1}{2} \right\rceil } \otimes G_{\left\lfloor \frac{t-1}{2} \right\rfloor }\right) ^{2} \textcircled {Z}H . \end{aligned}$$

From Lemma 18 it is easily seen that $G_t$ is a $[D^t,{\varDelta }^2,\lambda _t]$-graph with $\lambda _t$ defined by

$$\begin{aligned} \begin{aligned} \lambda _1&= \lambda ^2,&\\ \lambda _2&= \lambda ,&\\ \lambda _{2t-1}&= \lambda + \lambda _{t-1}^2,&\qquad \text {for }t&=2,3\ldots ,\text { and}\\ \lambda _{2t}&= \max (\lambda + \lambda _t^2, \lambda + \lambda _{t-1}^2),&\qquad \text {for }t&=2,3,\ldots . \end{aligned} \end{aligned}$$

### **Lemma 19**

(Reingold et al. [34, Theorem 3.3]) *The rotation map* $\mathrm {Rot}_{G_t}$ *can be computed in time* $\mathrm {poly}(t,\log D)$ *and by making* $\mathrm {poly}(t)$ *evaluations of* $\mathrm {Rot}_H$.

### **Lemma 20**

*If* $0\le \lambda \le 1/4$ *then* $\lambda _t \le \lambda + 4\lambda ^2$ *for all* $t\ge 1$.

### *Proof*

The conclusion is immediate for $t\le 2$. So suppose that the conclusion holds up to $2t-2$. We need to show that the conclusion holds for $\lambda _{2t-1}$ and $\lambda _{2t}$. By induction, it suffices to show that

$$\begin{aligned} \lambda _{2t-1} \le \lambda + (\lambda + 4\lambda ^2)^2 \le \lambda + 4\lambda ^{2}. \end{aligned}$$

Observing that $\lambda ^2 + 8\lambda ^3 +16\lambda ^4 \le 4 \lambda ^2$ holds for $0\le \lambda \le 1/4$ yields the desired conclusion. The proof for $\lambda _{2t}$ is identical. $\square$

Finally, we construct the expanders that we require in the manuscript proper.

### **Lemma 21**

(Lemma 2 stated with normalized eigenvalue notation) *For all integers* $t\ge 1$ *and* $b\ge 10$ *there exists a* $[2^{16bt},2^{4b},16\cdot 2^{-b}]$-*graph whose rotation map can be evaluated in time* $\mathrm {poly}(b,t)$.

### *Proof*

Take $q=2^b$ and $d=15$ in Proposition 5.3 of Reingold et al. [34] to obtain a $[2^{16b},2^{2b},15\cdot 2^{-b}]$-graph *H* whose rotation map can be computed in time $\mathrm {poly}(b)$. (Indeed, observe that an irreducible polynomial to perform the required arithmetic in the finite field of order $2^b$ can be constructed in deterministic time $\mathrm {poly}(b)$ by an algorithm of Shoup [35].) Let us study the sequence $G_t$ given by (36). The time complexity of the rotation map follows immediately from Lemma 19. Since $b\ge 10$, Lemma 20 gives that $\lambda _t\le \lambda + 4\lambda ^{2}$ for all $t \ge 1$. Take $\lambda =15\cdot 2^{-b}$ and observe that since $b\ge 10$ we have $2^{-b}<1/900$. Thus, $\lambda _t\le 15\cdot 2^{-b} + 4 (15 \cdot 2^{-b})^2 = 15 \cdot 2^{-b} + 900 \cdot 2^{-2b} \le 16 \cdot 2^{-b}$. $\square$

## Footnotes

1 The dimension is reduced by subsampling because the full $d^p$-dimensional Kronecker power is too large to be manipulated explicitly to yield subquadratic running times.

2 In comparison, a weaker form of explicitness could require, for example, that there exists a deterministic algorithm that computes the entire vector *f*(*x*) from a given *x* in time $D\cdot \mathrm {poly}(\log D,p)$.

3 The technical constraint $c_1n^{-c_2}\le \tau$ only affects inputs where the dimension *d* grows essentially as a root function of *n* since $\tau \ge 1/d$.

4 Actual *Ramanujan graphs* (see [18, 26]) would give somewhat stronger concentration $\lambda /{\varDelta }=O({\varDelta }^{-1/2})$ and hence improved constants in (4). However, we are not aware of a sufficiently fine-grained family of explicit Ramanujan graphs to comfortably support successive squaring.

5 Observe that from an information-theoretic perspective it is a positive-but-negligible-probability event that the drawn examples do not uniquely identify *S*.

6 *Caveat.* Reingold et al. [34] work with eigenvalues of the *normalized* adjacency matrix (with $|\lambda _1|=1$) whereas we follow Hoory et al. [18] and work with unnormalized adjacency matrices (with $|\lambda _1|={\varDelta }$) in the manuscript proper. “Appendix” works with normalized adjacency matrices for compatibility with Reingold et al. [34].
